# Assessment of suitable reference genes for RT–qPCR studies in chronic rhinosinusitis

**DOI:** 10.1038/s41598-018-19834-9

**Published:** 2018-01-25

**Authors:** Tsuguhisa Nakayama, Naoko Okada, Mamoru Yoshikawa, Daiya Asaka, Akihito Kuboki, Hiromi Kojima, Yasuhiro Tanaka, Shin-ichi Haruna

**Affiliations:** 1 Department of Otorhinolaryngology, Jikei University School of Medicine, Tokyo, Japan; 2 Department of Allergy and Immunology, National Research Institute of Child Health and Development, Tokyo, Japan; 3 Department of Otorhinolaryngology, Toho University, Tokyo, Japan; 4 Department of Otorhinolaryngology Head and Neck Surgery, Dokkyo Medical University Koshigaya Hospital, Saitama, Japan; 5 Department of Otorhinolaryngology Head and Neck Surgery, Dokkyo Medical University, Tochigi, Japan

## Abstract

Reverse transcription–quantitative polymerase chain reaction is a valuable and reliable method for gene quantification. Target gene expression is usually quantified by normalization using reference genes (RGs), and accurate normalization is critical for producing reliable data. However, stable RGs in nasal polyps and sinonasal tissues from patients with chronic rhinosinusitis (CRS) have not been well investigated. Here, we used a two-stage study design to identify stable RGs. We assessed the stability of 15 commonly used candidate RGs using five programs—geNorm, NormFinder, BestKeeper, ΔCT, and RefFinder. Ribosomal protein lateral stalk subunit P1 (*RPLP1*) and ribosomal protein lateral stalk subunit P0 (*RPLP0*) were the two most stable RGs in the first stage of the study, and these results were validated in the second stage. The commonly used RGs β-actin (*ACTB*) and glyceraldehyde 3-phosphate dehydrogenase (*GAPDH*) were unstable according to all of the algorithms used. The findings were further validated via relative quantification of *IL-5, CCL11, IFN-γ*, and *IL-17A* using the stable and unstable RGs. The relative expression levels varied greatly according to normalization with the selected RGs. Appropriate selection of stable RGs will allow more accurate determination of target gene expression levels in patients with CRS.

## Introduction

Reverse transcription–quantitative polymerase chain reaction (RT–qPCR) is routinely used for gene expression analysis. It provides a sensitive and reliable method for quantification of gene expression because of its wide range, high throughput, accurate quantification, and low cost^1–5^. However, the accuracy of the results may be affected by several factors related to biological and technical variations during the RNA extraction, reverse transcription, and RT–qPCR steps. Normalization is an essential component of reliable RT–qPCR analysis because it controls for these variations and thus allows comparison of gene expression levels among different samples^6^. An ideal reference gene (RG) must be stably expressed, non-regulated, and unaffected by biological or experimental conditions^7,8^. Selection of unstable RGs for normalization may lead to serious misinterpretations regarding the target gene of interest.

Chronic rhinosinusitis (CRS) is defined as nasal and sinonasal inflammation persisting for more than 12 weeks with two or more of the following typical symptoms: nasal obstruction, anterior/posterior nasal discharge, facial pain, or decreased sense of smell^9,10^. Although the pathophysiology of CRS has not been well elucidated, numerous studies have been published to date that have revealed some of the underlying mechanism. In these previous studies of CRS, traditional RGs such as β-actin (*ACTB*), glyceraldehyde-3-phosphate hydrogenase (*GAPDH*), and β-glucuronidase (*GUSB*) were used for RT–qPCR normalization^11–14^. However, suitable RGs for gene expression studies in CRS have not been well described. We therefore applied a two-stage study design and investigated the stability of potential RGs in nasal polyps and sinonasal tissues to determine appropriate RGs for analysing target gene expression levels in CRS.

In this study we examined the expression levels of the 15 RGs provided in the Human Housekeeping Gene Primer Set (Takara, Shiga, Japan) (Table 1) in 39 patients in the first stage of the study, and in 36 patients in the second stage. We analysed the stability of the 15 RGs using five different algorithms—geNorm^7^, Normfinder^15^, BestKeeper^16^, ΔCT^17^, and RefFinder^18^. Moreover, to validate the identified RGs, the relative expressions level of interleukin (IL)-5, interferon (IFN)-γ, and IL-17A, which are the major Th1/Th2/Th17 cell cytokines, and CCL11 which is the eosinophil chemoattractant chemokine were evaluated as target genes using the most stable RGs and conventional, less stable RGs for normalization.

**Table 1:** Reference genes evaluated in this study.

Symbol	Protein name	Accession number	Chromosome location	Amplicon length (bp)
*ACTB*	β-actin	NM_001101	7p22	186
*ATP5F1*	ATP synthase, H^+^ transporting, mitochondrial Fo complex subunit B1	NM_001688	1p13.2	142
*B2M*	β2-microglobulin	NM_004048	15q21.1	194
*GAPDH*	Glyceraldehyde 3-phosphate dehydrogenase	NM_002046	12p13	138
*GUSB*	β-glucuronidase	NM_000181	7q21.11	75
*HPRT1*	Hypoxanthine phosphoribosyltransferase 1	NM_000194	Xq26.1	131
*PGK1*	Phosphoglycerate kinase 1	NM_000291	Xq13.3	94
*PPIA*	Peptidylprolyl isomerase A	NM_021130	7p13	200
*RPLP0*	Ribosomal protein lateral stalk subunit P0	NM_053275	12q24.2	108
*RPLP1*	Ribosomal protein lateral stalk subunit P1	NM_213725	15q22	166
*RPLP2*	Ribosomal protein lateral stalk subunit P2	NM_001004	11p15.5	92
*RPS18*	Ribosomal protein S18	NM_022551	6p21.3	89
*TBP*	TATA box-binding protein	NM_003194	6q27	170
*TFRC*	Transferrin receptor	NM_003234	3q29	194
*YWHAZ*	Tyrosine 3-monooxygenase/tryptophan 5-monoxygenase activation protein, ζ-polypeptide	NM_145690	8q23.1	194

## Results

### Reference gene expression profiles

The PCR efficiency of each RG is shown in Supplementary Table S1. The slopes of the standard curves ranged from −3.280 to −3.107, the efficiencies from 101.8 to 109.8%, the correlation coefficients (R^2^) from 0.975 to 0.999, and the intercepts from 23.812 to 33.082. The melting curves revealed single peaks and no signal was detected in the negative controls for all primer pairs.

The quantification cycle (Cq) values indicated a wide range of expression levels from 15.28–32.08 in all samples (Supplementary Fig. S1). The high-abundance genes were *RPLP1*, *RPS18*, *RPLP2*, *B2M*, and *ACTB*, with mean Cq values of 16.43, 17.33, 17.51, 17.51, and 17.56, respectively. The low-abundance genes were *TBP*, *TFRC*, *HPRT1*, *GUSB*, and *PGK1*, with mean Cq values of 25.34, 24.06, 24.02, 23.30, and 23.01, respectively. The Cq values in all samples were less than 35.

### Analysis of gene expression stability

To determine the stability of RGs, the 15 RGs were analysed using geNorm^7^, Normfinder^15^, BestKeeper^16^, and ΔCT^17^. RefFinder^18^, a web based comprehensive evaluation platform, was then used to calculate an overall final ranking based on the results from the above four different algorithms. To evaluate anatomical variations in expression, we divided the 39 patients in the first population into nasal polyp, uncinate process, and control groups. In the second stage of the study, we analysed an independent group of 36 CRS patients to validate the results of the first stage. Additionally, we further classified CRS with nasal polyps (CRSwNP) into eosinophilic CRSwNP (ECRSwNP) and non-eosinophilic CRSwNP (NECRSwNP) according to previously published criteria^19^ to evaluate the influence of eosinophilic inflammation. The patient characteristics are shown in Supplementary Table S2.

#### geNorm analysis

**Figure 1:**
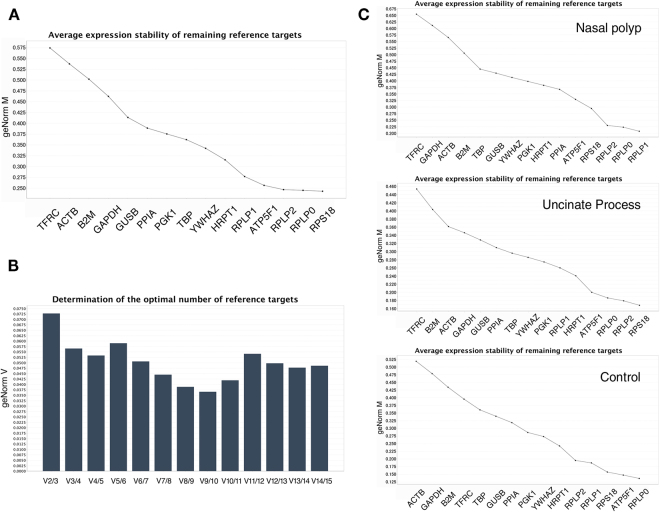
Stability ranking of candidate reference genes by geNorm in the first stage of the study. (**A**) Gene expression stability (geNorm M) in all samples. Least stable to the left and most stable to the right. (**B**) Determination of the optimal number of reference genes. The V_2/3_ value was below the 0.15 threshold and the optimal number of reference genes was two. (**C**) Gene expression stability (geNorm M) in subgroups.

**Figure 2:**
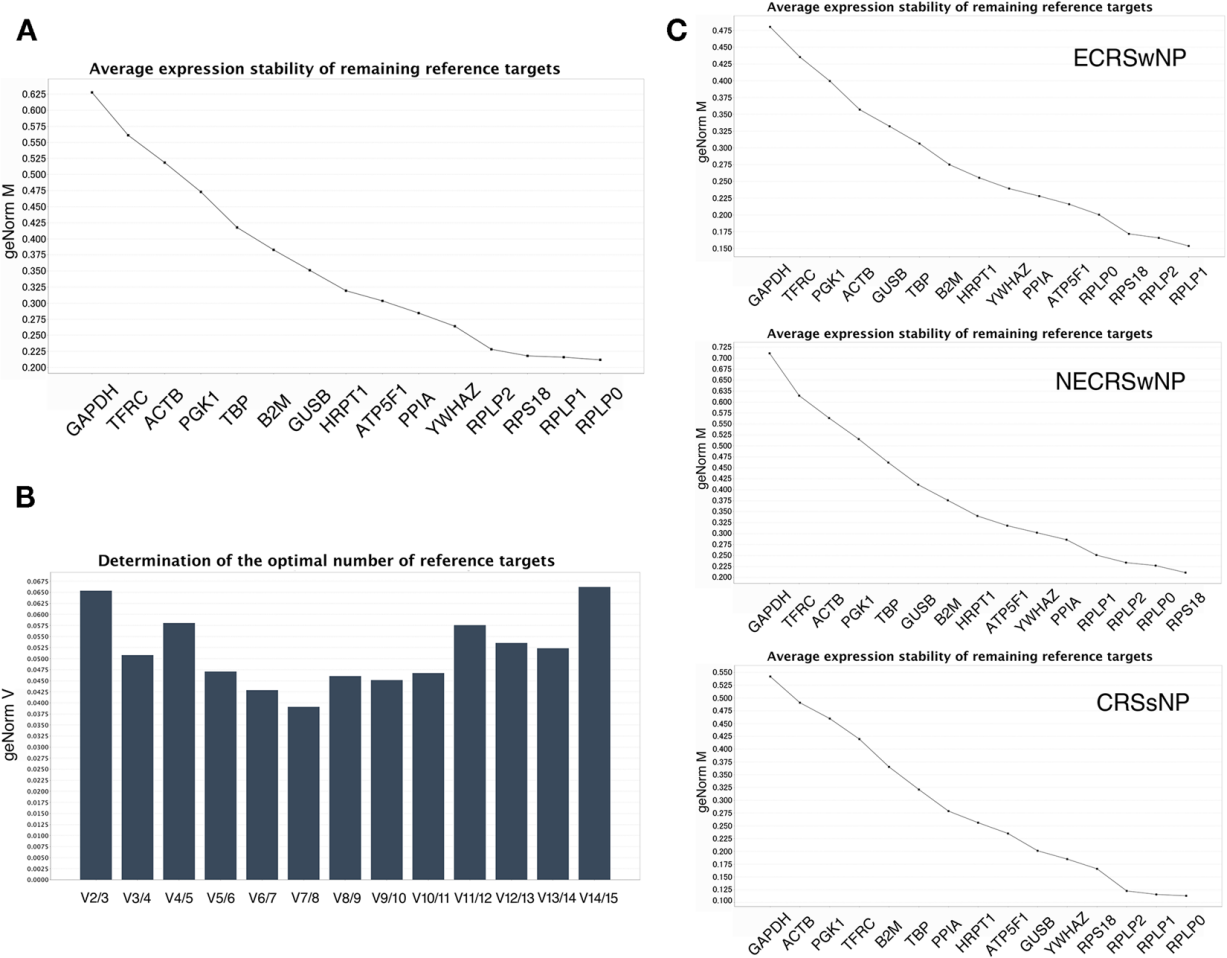
Stability ranking of candidate reference genes by geNorm in the second stage of the study. (**A**) Gene expression stability (geNorm M) in all samples. (**B**) Determination of the optimal number of reference genes. The V_2/3_ value was below the 0.15 threshold. (**C**) Gene expression stability (geNorm M) in subgroups.

According to the analysis of all of the samples in the first stage, *RPS18* had the lowest expression stability (M value) that means the most stable gene expression, followed by *RPLP0, RPLP2, ATP5F1*, and *RPLP1* (Fig. 1A). *TFRC*, *ACTB*, *B2M, GAPDH*, and *GUSB* were the five least stable genes. Of these RGs, *RPS18*, *RPLP0*, and *RPLP1* were validated as being among the five most stable RGs in the second stage of the study, and *TFRC*, *ACTB*, and *GAPDH* were validated as being among the five least stable RGs (Fig. 2A). The optimal number of reference genes was also determined using geNorm. The V_2/3_ value was below the threshold of 0.15 in all samples, indicating that two genes were sufficient for normalization (Figs 1B and 2B). In the subgroup analyses, *RPLP0, RPLP2, and RPS18* showed the highest stability in all tissues in both stages of the study (Figs 1C and 2C). The V_2/3_ value was below the threshold of 0.15 in each subgroup.

#### NormFinder analysis

We also calculated stability values using NormFinder software. The ranking of the five most stable candidate genes is shown in Table 2. Based on analysis of all the samples, *TBP* was the most stable RG, followed by *PGK1*, *PPIA*, *RPLP1*, and *HPRT1*, while *TFRC*, *B2M*, *ACTB, GAPDH*, and *GUSB* were the least stable genes. In the second study stage*, PPIA* and *RPLP1* were validated as being among the five most stable RGs and *TFRC*, *B2M*, *ACTB*, and *GAPDH* as the least stable RGs.

**Table 2:** Stability values of the five most stably expressed reference genes according to NormFinder.

Ranking	Total		Nasal polyps		Uncinate process		Control	
	Gene	Stability value	Gene	Stability value	Gene	Stability value	Gene	Stability value
1^st^ population								
1	*TBP*	0.254	*TBP*	0.264	*PPIA*	0.109	*PGK1*	0.142
2	*PGK1*	0.259	*PGK1*	0.308	*HPRT1*	0.132	*RPLP0*	0.208
3	*PPIA*	0.262	*RPLP0*	0.311	*PGK1*	0.146	*ATP5F1*	0.214
4	*RPLP1*	0.267	*GUSB*	0.315	*RPLP2*	0.193	*YWHAZ*	0.221
5	*HPRT1*	0.270	*RPLP1*	0.318	*YWHAZ*	0.238	*RPLP2*	0.241
2^nd^ population								
1	*RPLP0*	0.208	*RPLP0*	0.103	*RPLP0*	0.248	*PPIA*	0.037
2	*PPIA*	0.234	*ATP5F1*	0.142	*YWHAZ*	0.292	*RPS18*	0.138
3	*YWHAZ*	0.238	*PPIA*	0.143	*PPIA*	0.315	*RPLP0*	0.226
4	*RPS18*	0.263	*YWHAZ*	0.164	*RPS18*	0.336	*YWHAZ*	0.230
5	*RPLP1*	0.276	*RPLP1*	0.207	*RPLP1*	0.348	*GUSB*	0.235

#### BestKeeper analysis

BestKeeper identified the five most stable RGs listed in Table 3. *RPLP2*, *ATP5F1*, *RPS18*, and *RPLP0* were validated as being among the five most stable genes in the second step. *TFRC*, *ACTB, GAPDH*, and *TBP* were validated at the least stable RGs.

**Table 3:** Stability values of the five most stably expressed reference genes according to BestKeeper.

Ranking	Total			Nasal polyp			Uncinate process			Control		
	Gene	SD	CV	Gene	SD	CV	Gene	SD	CV	Gene	SD	CV
1^st^ population												
1	*RPLP2*	0.30	1.73	*ATP5F1*	0.29	1.37	*GUSB*	0.21	0.93	*RPLP2*	0.27	1.55
2	*GUSB*	0.31	1.34	*RPS18*	0.29	1.73	*RPLP2*	0.26	1.48	*PPIA*	0.28	1.51
3	*ATP5F1*	0.34	1.60	*RPLP2*	0.29	1.70	*ATP5F1*	0.26	1.21	*RPLP1*	0.28	1.71
4	*RPS18*	0.34	2.00	*HPRT1*	0.34	1.45	*PGK1*	0.29	1.26	*GUSB*	0.30	1.31
5	*RPLP0*	0.38	2.13	*RPLP1*	0.39	2.45	*HPRT1*	0.29	1.22	*RPLP0*	0.31	1.73
2^nd^ population												
1	*RPLP2*	0.28	1.30	*B2M*	0.16	0.75	*RPLP2*	0.18	0.85	*RPLP0*	0.24	1.09
2	*RPLP0*	0.29	1.34	*HPRT1*	0.22	0.80	*RPS18*	0.26	1.24	*ATP5F1*	0.27	0.77
3	*RPS18*	0.32	1.50	*RPLP0*	0.24	1.13	*RPLP0*	0.29	1.37	*RPLP2*	0.30	1.41
4	*RPLP1*	0.33	1.65	*ATP5F1*	0.27	1.07	*RPLP1*	0.31	1.56	*YWHAZ*	0.30	1.25
5	*ATP5F1*	0.34	1.35	*PPIA*	0.27	1.24	*ATP5F1*	0.36	1.44	*B2M*	0.31	1.45

#### ΔCT analysis

The RG stability according to ΔCT analysis is shown in Table 4. Among all of the samples, *RPLP1*, *PPIA*, *ATP5F1*, *RPLP0*, and *HPRT1* were the most stable RGs, while *TFRC*, *B2M*, *ACTB*, *GAPDH*, and *GUSB* were the least stable RGs. In the second stage of the study, *RPLP1*, *PPIA*, and *RPLP0* were validated as having high stability and *TFRC*, *B2M*, *ACTB*, *GAPDH* as having lower stability.

**Table 4:** Stability values of the five most stably expressed reference genes according to ΔCT analysis.

Ranking	Total		Nasal polyps		Uncinate process		Control	
	Gene	SD	Gene	SD	Gene	SD	Gene	SD
1^st^ population								
1	*RPLP1*	0.48	*ATP5F1*	0.54	*PPIA*	0.35	*RPLP0*	0.37
2	*PPIA*	0.48	*RPLP0*	0.54	*HPRT1*	0.36	*ATP5F1*	0.38
3	*ATP5F1*	0.48	*RPLP1*	0.55	*RPLP2*	0.36	*PGK1*	0.39
4	*RPLP0*	0.48	*RPLP2*	0.55	*PGK1*	0.37	*RPLP2*	0.40
5	*HPRT1*	0.48	*PPIA*	0.55	*RPS18*	0.38	*RPS18*	0.41
2^nd^ population								
1	*RPLP0*	0.47	*RPLP0*	0.35	*RPLP0*	0.53	*RPS18*	0.41
2	*YWHAZ*	0.49	*ATP5F1*	0.37	*YWHAZ*	0.55	*PPIA*	0.42
3	*RPS18*	0.50	*PPIA*	0.37	*PPIA*	0.55	*RPLP0*	0.42
4	*RPLP1*	0.50	*RPLP1*	0.39	*RPLP1*	0.58	*YWHAZ*	0.43
5	*PPIA*	0.50	*YWHAZ*	0.39	*RPS18*	0.58	*RPLP1*	0.45

#### RefFinder analysis

An overall final ranking was calculated based on the rankings from the previous four different programs, and is shown in Table 5. The comprehensive ranking from the most to the least stable expression is as follows: *RPLP1*, *RPLP0*, *RPLP2*, *PPIA*, *ATP5F1*, *RPS18*, *TBP*, *PGK1*, *HPRT1*, *GUSB*, *YWHAZ*, *GAPDH*, *ACTB*, *B2M*, and *TFRC*. In the second stage of the study, *RPLP1*, *RPLP0*, and *RPLP2* were validated as being among the five most stable RGs, whereas *TFRC*, *ACTB*, and *GAPDH* were validated as being among the least stable RGs. In the subgroup analyses, *RPLP0* was among the two most stable RGs in most of the subgroups, but showed moderate stability in the uncinate process subgroup in the first study stage. *RPLP2* showed high stability in all of the subgroups. Conversely, *TFRC*, *ACTB*, and *GAPDH* were the least stable RGs in all of the subgroups. We were not able to detect any apparent trends associated with different anatomical sites or inflammatory differences.

**Table 5:** Expression stability values of reference genes according to RefFinder.

Ranking	Total		Nasal polyp		Uncinate process		Control	
	Gene	Geometric value	Gene	Geometric value	Gene	Geometric value	Gene	Geometric value
1^st^ population								
1	*RPLP1*	3.31	*ATP5F1*	2.51	*RPLP2*	2.21	*RPLP0*	1.78
2	*RPLP0*	3.56	*RPLP0*	2.55	*PPIA*	2.71	*ATP5F1*	2.45
3	*RPLP2*	3.83	*RPLP1*	2.94	*HPRT1*	3.44	*RPLP2*	3.31
4	*PPIA*	3.98	*RPLP2*	3.98	*RPS18*	3.81	*PGK1*	3.83
5	*ATP5F1*	3.98	*TBP*	5.19	*GUSB*	4.09	*RPLP1*	5.01
6	*RPS18*	4.47	*RPS18*	5.58	*PGK1*	4.43	*PPIA*	5.03
7	*TBP*	5.27	*PPIA*	5.73	*ATP5F1*	6.03	*RPS18*	5.38
8	*PGK1*	5.73	*PGK1*	5.79	*RPLP0*	7.00	*YWHAZ*	7.09
9	*HPRT1*	6.12	*HPRT1*	6.90	*YWHAZ*	7.58	*HPRT1*	8.21
10	*GUSB*	7.18	*GUSB*	7.11	*RPLP1*	7.64	*GUSB*	8.34
11	*YWHAZ*	7.90	*YWHAZ*	9.49	*TBP*	10.69	*TBP*	9.23
12	*GAPDH*	12.24	*ACTB*	12.49	*GAPDH*	11.29	*TFRC*	12.47
13	*ACTB*	13.24	*B2M*	12.96	*ACTB*	12.49	*GAPDH*	12.98
14	*B2M*	13.47	*GAPDH*	13.49	*TFRC*	14.00	*B2M*	13.49
15	*TFRC*	15.00	*TFRC*	15.00	*B2M*	15.00	*ACTB*	15.00
2^nd^ population								
1	*RPLP0*	1.19	*RPLP0*	1.86	*RPLP0*	1.32	*RPLP0*	2.28
2	*RPLP1*	2.99	*ATP5F1*	2.99	*RPS18*	2.51	*RPS18*	2.91
3	*RPS18*	3.22	*RPLP1*	3.56	*RPLP2*	3.60	*PPIA*	3.44
4	*RPLP2*	3.60	*PPIA*	4.05	*YWHAZ*	3.72	*RPLP2*	3.48
5	*YWHAZ*	4.16	*RPLP2*	4.74	*PPIA*	4.05	*RPLP1*	3.66
6	*PPIA*	4.82	*B2M*	5.20	*RPLP1*	4.23	*YWHAZ*	4.23
7	*HPRT1*	7.20	*RPS18*	5.24	*ATP5F1*	6.77	*GUSB*	6.16
8	*ATPF1*	7.27	*HPRT1*	5.47	*GUSB*	7.84	*ATP5F1*	6.51
9	*GUSB*	8.21	*YWHAZ*	6.12	*HPRT1*	8.49	*HPRT1*	7.97
10	*B2M*	9.23	*GUSB*	9.87	*B2M*	8.91	*TBP*	9.43
11	*TBP*	10.22	*TBP*	10.74	*TBP*	11.00	*B2M*	9.82
12	*PGK1*	12.24	*ACTB*	10.95	*PGL1*	12.24	*ACTB*	11.74
13	*ACTB*	12.74	*PGK1*	13.00	*ACTB*	12.74	*PGK1*	12.22
14	*TFRC*	14.00	*TFRC*	14.00	*TFRC*	14.00	*TFRC*	14.00
15	*GAPDH*	15.00	*GAPDH*	15.00	*GAPDH*	15.00	*GAPDH*	15.00

### Influence of reference gene choice on the relative expression of target mRNA

**Figure 3:**
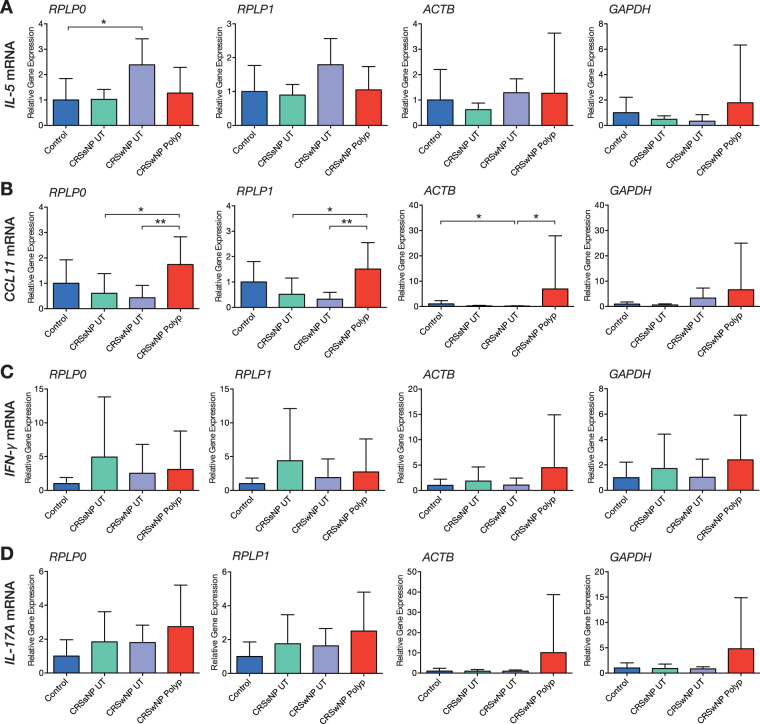
Effect of reference gene selection on relative quantification of *IL-5, CCL11, IFN-γ, and IL-17A mRNA* expression. Error bars represent standard deviation. Kruskal–Wallis with post-hoc Dunn’s multiple comparison tests were used. *p < 0.05, **p < 0.01.

To evaluate the influence of the RGs, the expression patterns of *IL-5, CCL11, IFN-γ*, and *IL-17A* were evaluated because CRS showed mixed enhanced Th1/Th2/Th17 reactions^14^. We chose the most stable RGs, *RPLP0* and *RPLP1*, and conventional, commonly used, but less stable RGs, *ACTB* and *GAPDH*, for normalization. The gene expressions were determined in the population that were analysed in the first part of the study. RG selection affected *IL-5* gene expression patterns between the subgroups as follows: *RPLP0* (*p* = 0.0181) and *RPLP1* (*p* = 0.0503), but *ACTB* (*p* = 0.0598) and *GAPDH* (*p* = 0.1675), assessed by Kruskal–Wallis test (Fig. 3A). *CCL11* gene expression patterns were also affected by RGs as follows: *RPLP0* (*p* = 0.0042), *RPLP1* (*p* = 0.0026), and *ACTB* (*p* = 0.0029), but *GAPDH* (*p* = 0.1390) (Fig. 3B). However, the expression pattern using *ACTB* was different from using *RPLP0* and *RPLP1*. Although *IFN-γ* and *IL-17A* did not show the differences, the genes exhibited similar expression trends when we normalized using *RPLP0* and *RPLP1*. Conversely, when *ACTB* and *GAPDH* were used, particularly wide variation in gene expressions was observed in nasal polyps from the CRSwNP group compared to the other RGs (Fig. 3C,D), and the trend was seen in all the four genes.

## Discussion

Here, we investigated the stability of candidate reference genes for RT–qPCR in nasal polyps and sinonasal tissues using a two-stage study design. Biological and experimental errors introduced throughout the RT–qPCR process need to be accounted for^5,6^, and normalization using an RG is a simple and popular method of internally controlling for such errors, as well as controlling for different input RNA amounts during the reverse transcription step^5^.

High RNA integrity and purity are critical for obtaining meaningful and reliable gene expression data and ensuring reproducibility of results^2,20^. Poor RNA integrity may generate misleading differences in gene expression measurements^1,20^. Stable RGs in nasal polyps and sinonasal tissues differ between intact and degraded RNA samples^21^. In this study, we chose samples with RNA integrity number (RIN) values ≥7 to avoid false results.

We used four different programs, geNorm, NormFinder, BestKeeper, and ΔCT to identify stable RGs. Unfortunately, we did not identify any RGs that were universally stable across these four programs. The discrepancies in the results occurred because the different programs use different algorithms, for example a pairwise comparison or a model-based approach^7,15–17^. To overcome the discrepancies and obtain a final ranking, we used RefFinder software. Two RGs, *RPLP1* and *RPLP0*, were identified as the two most stable RGs in the first stage of the study, and this was validated in the second stage. Our findings indicate that *RPLP0* and *RPLP1*, either singly or in combination, are suitable for normalizing gene expression in nasal polyp and sinonasal tissues. *RPLP0* has been used as an RG in previous CRS studies^22–24^. However, this study is the first to demonstrate its stability in nasal polyp and sinonasal tissues. Conversely, *TFRC*, *ACTB*, and *GAPDH* were among the five least stable genes throughout all of the algorithms, including RefFinder. Based on these results, these conventionally used RGs should not be used.

Narrower standard deviations and the different *p*-values in the gene expressions were revealed when we used the most stable RGs, *RPLP0* and *RPLP1*, for normalization, compared with when we used the commonly used, less stable genes *GAPDH* and *ACTB*. The selection of RGs could shift results from indicating significant differences to being non-significant, and vice-versa. Normalizing using *GAPDH*, especially, produced a different expression trend. *GAPDH* mRNA expression levels are known to differ between different tissues and between the same tissues in different individuals^25^. In this study, *GAPDH* showed lower stability in all of the anatomical sites and inflammation patterns studied compared to other RGs. It has been reported that *GAPDH* and *ACTB* were not suitable as RGs for quantitative analysis of gene expression in asthma, which has similar pathophysiology to CRS^26^. These results emphasise the importance of choosing stable RGs for normalization.

A large number of studies have investigated the validation of reference genes in many different tissues and cell types. However, to the best of our knowledge, few have examined the suitability of reference genes in CRS. Perez-Novo *et al*.^21^ investigated 16 samples, including degraded samples, from ethmoid and maxillary sinuses from patients with nasal polyps and CRS. In intact RNA, they found that the genes for hydroxymethyl-bilane synthase (*HMBS*) and succinate dehydrogenase complex, subunit A (*SDHA*) in CRS, and *ACTB* and *TBP* in nasal polyps were the most stable among nine candidate reference genes analysed using geNorm. *GUSB* and ATPase plasma membrane Ca^2+^ transporting 4 (*ATP2B4*) were identified as reliable genes for normalization of cystic fibrosis transmembrane conductance regulator (*CFTR)* gene expression in the nasal mucosa and nasal polyps in patients with cystic fibrosis^27^. These RGs differ from those identified in the current study. These differences may be attributed to differences in sample sizes, sampling location, differences in pathophysiology within CRS subgroups, or ethnic differences between Western and Japanese populations^28–30^.

## Conclusion

This study identified suitable RGs for normalizing target gene expression levels in nasal and sinus tissues using a two-stage study design. *RPLP0* and *RPLP1*, either singly or in combination, are suitable for normalizing gene expression in nasal and sinus tissues, whereas *TFRC*, *ACTB*, and *GAPDH* were less stable RGs according to all of the algorithms used. Use of appropriate reference genes will facilitate the generation of accurate, robust, and reproducible gene expression studies in CRS.

## Methods

### Patients

We prospectively enrolled 29 patients with CRS and 10 control patients at Jikei University School of Medicine, Dokkyo Medical University, and Dokkyo Medical University Koshigaya Hospital from February to November 2015 in the first stage of the study. For the second, validation stage of the study, we enrolled 36 patients with CRS at Toho University Ohashi Hospital from January 2015 to December 2016. The study was approved by the ethical committees of Jikei University School of Medicine, Dokkyo Medical University, Dokkyo Medical University Koshigaya Hospital, and Toho University Ohashi Hospital. We complied with the Declaration of Helsinki and relevant ethical regulations of each institution, and written informed consent was obtained from each patient. The diagnosis of CRS was made according to published guidelines^9,10^. Exclusion criteria were as follows: treatment with oral steroids or antimicrobial agents within 4 weeks before surgery; and unilateral disease, fungal disease, antrochoanal polyps, allergic fungal rhinosinusitis, or paranasal sinus cysts. Demographic and clinical characteristics were obtained from the patients prior to surgery, including age, sex, asthma status, and history of sinus surgery. Preoperative computed tomography scans were assessed according to the classification described by Lund and Mackay^31^. The preoperative polyp-grading system used a 5-point scale (score of 0–4) according to the recommended guidelines^32^. Blood samples were taken before surgery, and complete blood counts and serum total IgE levels were determined.

### Sampling and total RNA extraction

At the surgery, we removed nasal polyps from patients with CRS, and uncinate processes from CRS patients with/without nasal polyps and control patients. The control group consisted of 10 patients with pituitary tumours or anatomical variants, without endoscopic and radiological evidence of sinus disease, in the first population. Under 0-degree endoscope, the samples were obtained with a scalpel and Grunwald’s forceps without local anaesthesia instillation. The tissues were immediately immersed in RNA later (Ambion, Austin, TX, USA) and stored at 4 °C for 1–2 days, then at −80 °C until analysis.

We extracted RNA using NucleoSpin RNA (Macherey-Nagel, Düren, Germany) in the first stage of the study and an miRNeasy Mini Kit (Qiagen, Hilden, Germany) for the second stage, both according to the manufacturer’s instructions. The quality and quantity of the extracted RNA were determined by measuring the ratios of absorbance at 260/230 nm and 260/280 nm using a DeNovix DS-11 spectrophotometer (DeNovix, Wilmington, DE, USA) and a NanoDrop 2000 spectrophotometer (Thermo, Wilmington, DE, USA).

RNA integrity was confirmed with an RNA 6000 Nano Chip using an Agilent 2100 Electrophoresis Bioanalyzer (Agilent Technologies, Santa Clara, CA, USA). An RIN ≥7 was considered adequate for analysis, on a scale where 1 indicated the most degraded and 10 the most intact profile.

### qPCR

A sample of total RNA was reverse transcribed into cDNA using PrimeScript RT Master Mix (Takara, Shiga, Japan) in the first stage of the study and an iScript cDNA Synthesis kit (Bio-Rad, Hercules, USA) for the second stage, according to the manufacturers’ instructions. Reverse transcription was performed in a TaKaRa PCR Thermal Cycler Dice Gradient (Takara) and iCycler Thermal Cycle system (Bio-Rad). cDNA was stored at −20 °C until qPCR experiments were performed.

We examined the expression levels of the 15 reference genes provided in the Human Housekeeping Gene Primer Set (Takara) (Table 1). All of the PCR products ranged from 75 to 200 bases.

qPCR amplification reactions were performed using a Light Cycler 96 (Roche, Mannheim, Germany) for the first stage of the study and a CFX96 Touch Real-Time PCR Detection System (Bio-Rad) for the second. Amplifications were performed with 30 s enzyme activation at 95 °C, followed by 40 cycles of denaturation at 95 °C for 5 s, and then annealing/extension at 60 °C for 20 s. At the end of each run, melting curve analysis was performed from 65 °C to 95 °C. Briefly, 2 μl of cDNA equivalent to 10 ng of total RNA was used as a template in a total reaction volume of 10 μl containing 5 μl of SYBR Premix Ex Taq II (Takara), 200 nM of each primer, and RNase/DNase-free water.

The slope, efficiency, correlation coefficient (R^2^), and intercept of each primer pair were determined from the standard curve created using 5-point serial dilutions of cDNA template mixture, and analysed by qbase^plus^ (Biogazelle, Ghent, Belgium). The absorbance ratios (mean ± standard deviation) at 260/230 nm (2.07 ± 0.14) and 260/280 nm (2.13 ± 0.08) indicated that the RNA samples were pure and free of protein. The mean RIN values were 8.04 ± 0.58.

### Determination of gene expression levels based on different RGs

To confirm whether the identified RGs were stable, the most stable RGs and conventionally used but less stable RGs were used for normalization to calculate the relative expression levels of the target gene. The primers sequences were as follows: *IL-5*, forward; 5′-TGCCATCCCCACAGAAATTC-3′ and reverse, 5′-TGCCAAGGTCTCTTTCACCAA-3′, *CCL11*, forward; 5′-TCTGTGGCTGCTGCTCATAG-3′ and reverse, 5′-TGCCACTGGTGATTCTCCTG-3′, *IFN-γ*, forward; 5′-CAGGTCATTCAGATGTAGCGGA-3′ and reverse, 5′-TCTGTCACTCTCCTCTTTCCAAT-3′, and *IL-17A*, forward; 5′-TGGTGTCACTGCTACTGCTG-3′ and reverse, 5′-GCATCCTGGATTTCGTGGGA-3′.

### Statistical analysis

The stability of gene expression was analysed using geNorm^7^, Normfinder^15^, BestKeeper^16^, and ΔCT^17^. RefFinder^18^ was then used to calculate an overall final ranking based on the results from the above four different algorithms. Raw Cq values were analysed in qbase^plus^ and the expression stability (M value) of each reference gene was calculated as the average pairwise variation for the reference gene with all of the other genes. A low M value represented stable gene expression^7^. Additionally, the pairwise variation (V_n/n+1_) between the two sequential normalization factors (NF_n_ and NF_n+1_) was calculated to define the optimal number of reference genes. When the V value was below the cut-off of 0.15, it was not necessary to include additional reference genes^7^. The NormFinder add-in for Microsoft Excel was used to estimate both intragroup and intergroup variation for each candidate reference gene. These two variation values were combined to give a stability value, with the lowest value indicating the most stable expression^15^. BestKeeper calculated RG stability based on the standard deviation (SD) of Cq values; RGs with SD > 1 were excluded^16^. ΔCT generated ‘pair of genes’ comparisons between each RG and the other RGs within each sample and calculated the average SD against the other RGs^17^. Finally, based on the rankings of these four algorithms, RefFinder, a web based comprehensive evaluation platform, was used (http://www.leonxie.com/referencegene.php).

Statistical analysis was performed using IBM SPSS Statistics version 23 (IBM Corporation, Armonk, NY, USA) and graphs were produced using GraphPad Prism 6 for Mac OS X (GraphPad Software Inc., San Diego, CA, USA). Continuous and categorical data were compared among subgroups using Kruskal–Wallis and χ^2^ tests. The Kruskal–Wallis with post-hoc Dunn’s multiple comparison test was used for comparing more than two groups. Nasal polyp score was compared between two groups using Mann–Whitney U-tests. A value of *p* < 0.05 was considered to indicate a statistically significant difference.

### Data Availability Statement

All data generated or analysed during this study are included in this published article (and its supplementary information files).

## Electronic supplementary material

Supplementary Information
