# Supplementary material for: Assessment of suitable reference genes for RT–qPCR studies in chronic rhinosinusitis
Source: Sci Rep. 2018 Jan 25;8:1568. doi: 10.1038/s41598-018-19834-9 (PMC5785529; doi:10.1038/s41598-018-19834-9)
Supplement: Supplementary file 1 — Supplementary Information [file 41598_2018_19834_MOESM1_ESM.pdf]

Assessment of suitable reference genes for RT–qPCR studies in chronic rhinosinusitis

Tsuguhisa Nakayama, Naoko Okada, Mamoru Yoshikawa, Daiya Asaka, Akihito Kuboki, Hiromi Kojima, Yasuhiro Tanaka, Shin-ichi Haruna

Corresponding author: Tsuguhisa Nakayama

Supplementary information

**Variables**	**Slope**	**Efficiency**	**R^2**	**Intercept**
ACTB	-3.167	106.7	0.998	24.048
ATP5F1	-3.280	101.8	0.995	29.535
B2M	-3.167	106.9	0.997	25.409
GAPDH	-3.144	108.0	0.996	26.556
GUSB	-3.159	107.3	0.995	31.360
HPRT1	-3.122	109.1	0.996	31.260
PGK1	-3.200	105.4	0.993	30.202
PPIA	-3.156	107.4	0.994	25.494
RPLP0	-3.240	103.5	0.987	25.193
RPLP1	-3.107	109.8	0.994	23.812
RPLP2	-3.176	102.0	0.999	25.036
RPS18	-3.121	109.1	0.998	24.873
TBP	-3.235	103.8	0.975	33.082
TFRC	-3.219	104.4	0.989	30.780
YWHAZ	-3.259	102.3	0.998	28.498

Supplementary Table S1. qPCR parameters for the standard curve for each primer pair

	First stage					Second stage			
**Variables**	**NP CRSwNP n=11**	**UP CRSwNP n=10**	**UP CRSsNP n=8**	**UP Control n=10**	***p* value**	**NP ECRSwNP n=13**	**NP NECRSwNP n=15**	**UP CRSsNP n=8**	***p* value**
Age (mean ± SD)	51.9 ± 19.2	59.1 ± 12.6	50.9 ± 14.7	41.0 ± 11.2	0.086	53.8 ± 10.0	55.9 ± 18.8	51.1 ± 19.4	0.813
Sex, %female	18.2	10.0	50.0	20.0	0.218	61.5	26.7	37.5	0.169
CT score	14.8 ± 4.6	14.4 ± 3.1	9.8 ± 2.7	-	0.100	11.2 ± 6.0	10.7 ± 5.6	5.9 ± 3.3	0.047
Polyp Score	4.8 ± 2.0	4.1 ± 1.7	-	-	0.427	8.0 ± 6.6	3.1 ± 2.9	-	0.010
Asthma, n(%)	1 (9.1)	0 (0.0)	1 (12.5)	1 (10.0)	0.761	7 (53.8)	4 (26.7)	2 (25.0)	0.249
Previous surgery, n %)	1 (9.1)	0 (0.0)	0 (0.0)	0 (0.0)	0.455	4 (19.4)	2 (13.3)	1 (12.5)	0.434
History of smoking									
Current smoker, n (%)	3 (27.3)	1 (10.0)	1 (12.5)	1 (10.0)	0.639	0 (0.0)	2 (13.3)	1 (12.5)	0.396
Past smoker, n (%)	2 (18.2)	5 (50.0)	2 (25.0)	3 (30.0)	0.443	3 (23.1)	8 (53.5)	4 (50.0)	0.233
Blood test									
White blood cells (10^3/μl)	6.4 ± 1.3	5.6 ± 1.1	6.1 ± 1.9	5.8 ± 1.2	0.514	6.8 ± 2.2	6.0 ± 1.1	5.1 ± 1.5	0.086
Eosinophils (%)	5.9 ± 4.6	5.5 ± 2.5	6.3 ± 2.3	3.7 ± 2.4	0.272	7.7 ± 4.7	4.5 ± 2.5	5.1 ± 2.5	0.078
Total IgE (IU/ml)	197.3 ± 320.8	162.7 ± 188.2	218.1 ± 180.0	1383.1 ± 2460.0	0.717	153.1 ± 121.7	206.2 ± 240.4	439.7 ± 363.7	0.192

Supplementary Table S2. Demographic and clinical characteristics of the participants

NP = nasal polyp, UP = uncinate process, CRSwNP = chronic rhinosinusitis with nasal polyps, CRSsNP = chronic rhinosinusitis without nasal polyps, ECRSwNP = eosinophilic chronic rhinosinusitis with nasal polyps, NECRSwNP = non-eosinophilic chronic rhinosinusitis with nasal polyps, CT = computed tomography.

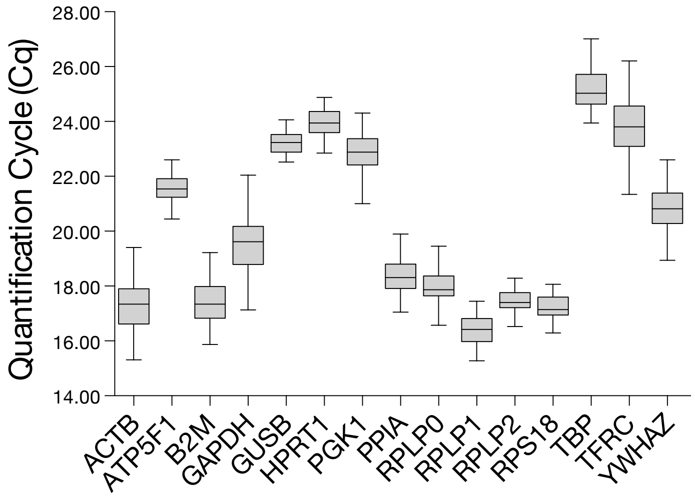

Supplementary Figure S1. Quantification cycle (Cq) values for candidate reference genes
